# Obesity and Dural Venous Sinus Thrombosis: An Unusual Presentation of an Uncommon Stroke

**DOI:** 10.7759/cureus.97922

**Published:** 2025-11-27

**Authors:** James Guider, Arshia Mahmoodi, Sofia Terner

**Affiliations:** 1 Physical Medicine and Rehabilitation, Rutgers University New Jersey Medical School, Newark, USA; 2 Internal Medicine, University of Buffalo, Buffalo, USA; 3 Internal Medicine, Overlook Medical Center, Summit, USA

**Keywords:** cerebral venous and dural sinus thrombosis, cerebro-vascular accident (stroke), comorbid obesity, dural venous sinus thrombosis, imaging findings, stroke risk factor

## Abstract

Dural venous sinus thrombosis (DVST) is a subset of cerebral venous sinus thrombosis (CVST) and is a rare diagnosis, typically observed in women or people with identifiable hypercoagulable risk factors. DVST can present similarly to other medical conditions, requiring neuroimaging for accurate identification and prompt treatment. Obesity, a rapidly growing global health problem with numerous health implications, is not well defined as being associated with DVST in the available medical literature. In this case report, we describe a male patient presenting with headache and vision changes, initially suspicious for idiopathic intracranial hypertension, ultimately diagnosed with DVST whose sole identifiable risk factor was obesity, representing a potential expansion of the current understanding of CVST risk stratification.

## Introduction

Cerebral venous sinus thrombosis (CVST) is a rare diagnosis, presenting with variable and often nonspecific complaints, ranging from the most common headache to altered mental status and seizures as the more severe. CVST represents only about 0.5% of all cerebrovascular disease cases globally [1], and the yearly incidence of CVST has been previously reported as 1.32 per 100,000 people (i.e., 0.00132%) [2], although this may represent an underestimation of the incidence of CVST due to nonspecific presenting symptoms and the need for advanced neuroimaging for detection. CVST is an occlusion of intracranial venous channels, including dural venous sinuses, cortical veins, and deep cerebral veins. Dural venous sinus thrombosis (DVST) is a specific subset of CVST. DVST occurs when thrombus formation obstructs venous drainage, potentially leading to increased intracranial pressure, venous infarction, hemorrhage, permanent neurologic deficits, and increased mortality if untreated. Idiopathic intracranial hypertension (IIH) has significant overlap with CVST in both clinical presentation as well as risk factors, which poses many diagnostic challenges. Obesity is well-documented to be a growing public health concern as a major contributor to morbidity and mortality. Obesity’s clinical relevance continues to surge, with models projecting that approximately one out of two Americans will be obese by 2030 [3]. Furthermore, obesity is known to promote a prothrombotic state through chronic inflammation and impaired fibrinolysis [4]. While obesity is a clear risk factor for IIH, which may present similarly to CVST, obesity's association with CVST is less apparent and lacks documented evidence of occurring in men.

This article was previously presented as a poster at the 2025 Association of Academic Physiatrists annual meeting on February 28, 2025.

## Case presentation

This case highlights DVST occurring in an obese male without other identifiable thrombophilic or systemic risk factors. A 40-year-old Caucasian male with a past medical history of hypertension and obesity (body mass index: 41) presented with a chief complaint of headache and vision changes for one month. The headache was described as sudden onset, pulsatile, non-radiating, localized to the frontal lobe region, exacerbated by Valsalva maneuver and positional changes, associated with pulsatile tinnitus, vomiting, and decreased vision bilaterally. Vision changes were characterized as tunnel vision, floaters, and flashing lights, regardless of the patient's position. The patient denied associated photophobia, weakness, behavioral changes, or prior history of headaches. Furthermore, the patient denied a history of recent trauma, infection, smoking, alcohol use, or family history of prothrombotic disorders.

The patient was evaluated as an outpatient prior to presentation, with a workup notable for an MRI of the brain, although without venous sequences, which was unremarkable, and an evaluation by an ophthalmologist, who identified bilateral papilledema and sent the patient to the Emergency Department. Initial vitals were temperature 36.8°C, blood pressure 151/83 mmHg, heart rate 100 bpm, SpO2 98% on room air. Neurologic examination demonstrated an alert and oriented patient with no meningeal signs and no apparent distress. Pupils were equal and reactive to light with normal eye movements and intact visual fields, fully intact cranial nerves, absence of sensory deficits, full 5/5 strength throughout all muscle groups, and normal coordination and gait. The remainder of the physical examination was within normal limits.

Laboratory tests, including complete blood count and comprehensive metabolic panel, were within normal limits. CT angiography of the head/neck with contrast showed no large vessel stenosis or occlusion; neither cord sign nor an empty delta sign was identified. IIH was suspected, and a lumbar puncture was performed, with an elevated opening pressure of 43 cm H₂O (normal value 10-25 cm H₂O in adults). Cerebral spinal fluid (CSF) cell count, differential, protein, and glucose levels were normal, and cultures were negative.

**Figure 1:**
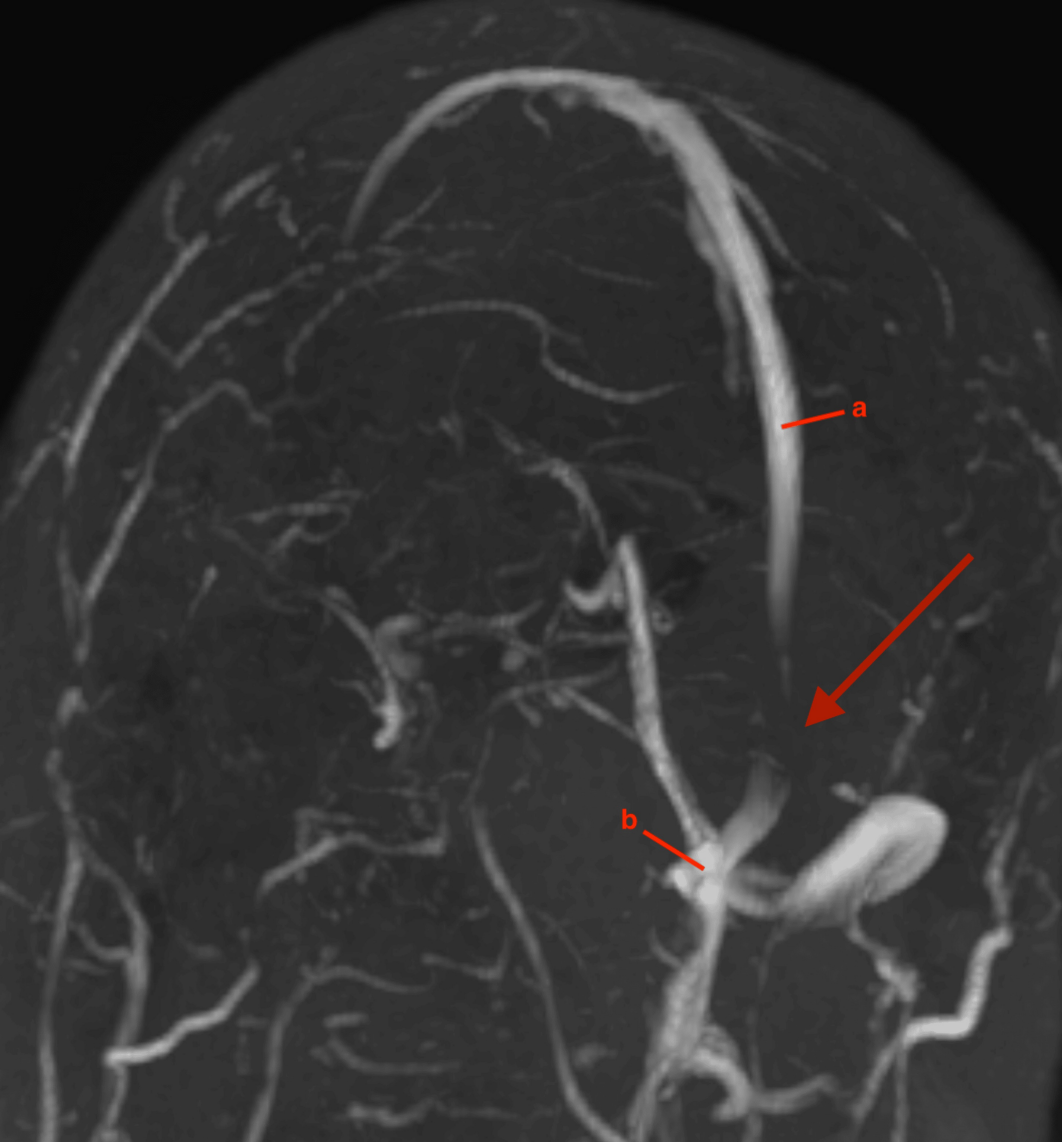
3D reconstruction of magnetic resonance venography with red arrow demonstrating a filling defect at the inferior aspect of superior sagittal sinus extending to the torcula, with absence of signal representing thrombosis. (a) Superior sagittal sinus. (b) Torcula.

**Figure 2:**
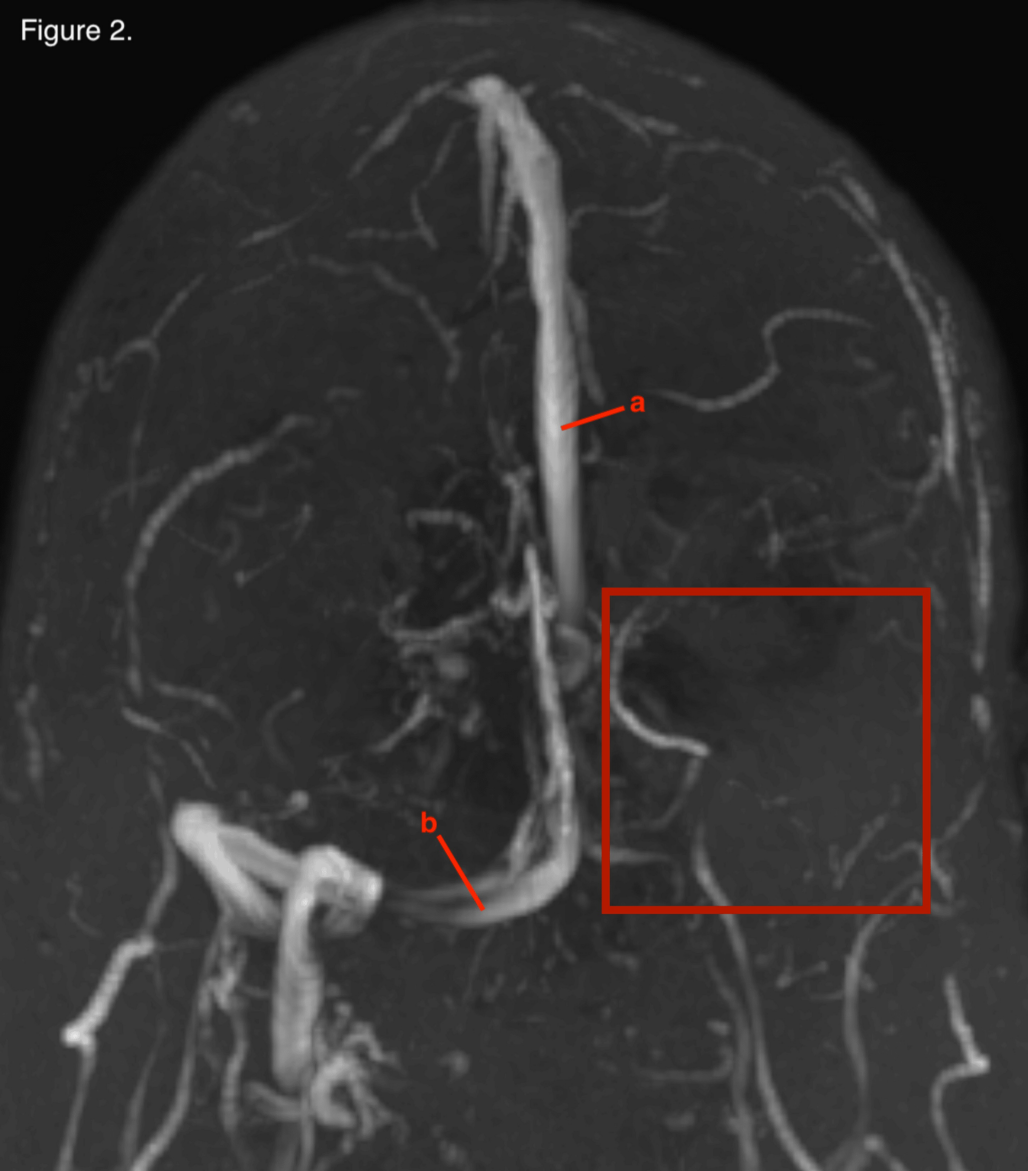
3D reconstruction of magnetic resonance venography with red box highlighting absence of left transverse sinus secondary to dural venous sinus thrombosis. (a) Superior sagittal sinus. (b) Contralateral transverse sinus.

Following lumbar puncture, the patient endorsed immediate relief of symptoms with resolution of vision changes and headaches. He was started on Acetazolamide for suspected IIH. The patient was sent for MR venogram (MRV) and MR arteriogram (MRA) of the head with and without contrast to rule out CVST. MRA was unremarkable. MRV showed a filling defect in the region of the inferior aspect of the superior sagittal sinus extending to the torcula (Figure 1), from the torcula into the transverse sinuses bilaterally, and from the left transverse sinus to the sigmoid sinus (Figure 2); these findings were consistent with DVST.

The patient was started on anticoagulation with Enoxaparin (1 mg/kg twice daily) for two days until discharge. Acetazolamide was continued for papilledema until clearance was obtained by Ophthalmology. On discharge, the patient was transitioned to Apixaban. Hypercoagulability workup with antithrombin III activity, protein C activity, factor V Leiden, prothrombin gene mutation, cardiolipin and beta 2 glycoprotein antibodies, and JAK2 mutation was negative. A CT of the chest, abdomen, and pelvis with contrast was obtained, which was negative for malignancy. At outpatient follow-up with hematology after hospital discharge, the patient reported he was symptom-free with a plan for six months of anticoagulation and repeat imaging after completion.

## Discussion

CVST is an uncommon diagnosis, and known risk factors include prothrombotic hematologic conditions (e.g., protein S deficiency, polycythemia vera, factor V Leiden deficiency), systemic illness (e.g., cancer, sepsis), local trauma (e.g., skull fractures, anatomic abnormalities), infections (e.g., mastoiditis, sinusitis), and hormonal etiologies (e.g., oral contraceptives (OCPs), pregnancy, hyperthyroidism). Obesity can promote a prothrombotic state through chronic inflammation, leading to impaired fibrinolysis and endothelial dysfunction [4]. Studies have highlighted obesity as a possible risk factor for CVST. For example, Zuurbier et al. investigated the association of obesity and CVST, finding CVST was only significantly associated with obesity in women on OCPs, but notably, there was no association in women not taking OCPs or in obese men [3]. This case challenges these findings and demonstrates an opportunity to further elucidate mechanisms by which sex, for example, sex-specific hormones, may modify the risk of CVST. Obesity can increase an individual's risk for a myriad of health problems. Particularly in the United States, the prevalence of obesity continues to increase [5]. As a result of this projected growth, it is imperative for clinicians to be aware of increased risks and associated complications of obesity.

IIH is also associated with obesity, particularly in females, although the underlying pathophysiologic mechanism for this association remains poorly understood [6]. The clinical presentation of CVST and IIH can often be indistinguishable, and usually requires neuroimaging such as MRI, MRV, or MRA to differentiate the two diagnoses [7]. A diagnosis of IIH, according to the modified Dandy criteria, requires symptoms of increased intracranial pressure, elevated intracranial pressure with normal CSF composition, no other neurologic abnormalities, no impaired consciousness, no other apparent cause of intracranial hypertension, and neuroimaging demonstrating no secondary etiology for increased intracranial hypertension [8]. The clinical presentation of this patient met some, but not all, of the modified Dandy criteria, which emphasizes the potential diagnostic overlap between IIH and CVST, necessitating MRV.

The current body of literature on CVST emphasizes its rarity, but in the majority of cases, conventionally accepted risk factors are identified. Kalita, Misra, and Singh evaluated risk factors for CVST and found that only 26 of 128 patients with CVST had unidentified risk factors [9], underscoring the importance of not dismissing obesity as a benign comorbidity in stroke evaluations. Other cases of CVST described in the literature without identifiable risk factors were limited by inability to obtain thorough history [10], or had later identified, albeit less understood, risk factor such as lupus-like anticoagulant [11], elevated plasminogen activator inhibitor 1 (PAI-1) levels [12], remote history of squamous cell carcinoma [13], nephrotic syndrome [14], or even COVID-19 infection [15]. Our patient's workup included limitations, such as a lack of a sleep study to investigate undiagnosed sleep apnea, an unknown history of prior COVID-19 infection, or a lack of measurement of PAI-1 and inflammatory cytokines, which could have revealed subclinical prothrombotic states. Additionally, the influence of pre-existing hypertension on the development of CVST in this patient is unclear. Despite these limitations, our patient had a thorough hematologic workup for thrombophilia, including hereditary causes, which failed to identify any clear predisposing risk factor, highlighting this unique case presentation.

## Conclusions

We describe a case of DVST occurring in a patient with a negative hematologic workup and without identifiable risk factors other than obesity. Obesity is a continually growing global epidemic, and further investigation into possible sequelae of obesity will be imperative to improve patient outcomes. While prior studies found no association between obesity and CVST in men, this case raises questions about whether obesity may contribute to CVST in specific circumstances. This case supplements available literature by identifying isolated obesity as a potential independent risk factor worth further epidemiologic investigation. It is also important to highlight the significance of neuroimaging to rule out secondary causes of IIH, particularly given the overlap in clinical presentation. In this case, lumbar puncture demonstrated increased opening pressure and was associated with immediate symptom improvement. If an MRV was not obtained after lumbar puncture, it is possible our patient’s diagnosis of DVST could have been missed or delayed, potentially contributing to increased morbidity. This emphasizes that clinicians must maintain a high index of suspicion for CVST in obese patients presenting with headache and papilledema, even in the absence of typical risk factors, and may consider MRV as first-line investigation if IIH or CVST is suspected. Clinicians should also be aware that obesity may increase the risk of CVST in a patient without conventional risk factors, and the importance of urgent neuroimaging when there is a concern for CVST.
